# Intermittent screening and treatment with dihydroartemisinin-piperaquine for the prevention of malaria in pregnancy: implementation feasibility in a routine healthcare system setting in western Kenya

**DOI:** 10.1186/s12936-020-03505-0

**Published:** 2020-11-25

**Authors:** Jenny Hill, Peter Ouma, Seth Oluoch, Jane Bruce, Simon Kariuki, Meghna Desai, Jayne Webster

**Affiliations:** 1 Department of Clinical Sciences, Liverpool School of Tropical Medicine, Liverpool, UK; 2 Kenya Medical Research Institute/Centre for Global Health Research, Kisumu, Kenya; 3 Centers for Disease Control and Prevention, Atlanta, GA USA; 4 Disease Control Department, London School of Tropical Medicine and Hygiene, London, UK

**Keywords:** Intermittent screening and treatment, Intermittent preventive treatment, Dihydroartemisinin-piperaquine, Health system delivery, Pregnant women, Health providers, Kenya

## Abstract

**Background:**

Intermittent preventive treatment in pregnancy (IPTp) with sulfadoxine-pyrimethamine (SP) is recommended for preventing malaria in pregnancy in areas of moderate-to-high transmission in sub-Saharan Africa. However, due to increasing parasite resistance to SP, research on alternative strategies is a priority. The study assessed the implementation feasibility of intermittent screening and treatment (ISTp) in the second and third trimester at antenatal care (ANC) with malaria rapid diagnostic tests (RDTs) and treatment of positive cases with dihydroartemisinin-piperaquine (DP) compared to IPTp-SP in western Kenya.

**Methods:**

A 10-month implementation study was conducted in 12 government health facilities in four sub-counties. Six health facilities were assigned to either ISTp-DP or IPTp-SP. Evaluation comprised of facility audits, ANC observations, and exit interviews. Intermediate and cumulative effectiveness analyses were performed on all processes involved in delivery of ISTp-DP including RDT proficiency and IPTp-SP ± directly observed therapy (DOT, standard of care). Logistic regression was used to identify predictors of receiving each intervention.

**Results:**

A total of 388 and 389 women were recruited in the ISTp-DP and IPTp-SP arms, respectively. For ISTp-DP, 90% (289/320) of eligible women received an RDT. Of 11% (32/289) who tested positive, 71% received the correct dose of DP and 31% the first dose by DOT, and only 6% were counselled on subsequent doses. Women making a sick visit and being tested in a facility with a resident microscopist were more likely to receive ISTp-DP (AOR 1.78, 95% CI 1.31, 2.41; and AOR 3.75, 95% CI 1.31, 2.40, respectively). For IPTp-SP, only 57% received a dose of SP by DOT. Payment for a laboratory test was independently associated with receipt of SP by DOT (AOR 6.43, 95% CI 2.07, 19.98).

**Conclusions:**

The findings indicate that the systems effectiveness of ANC clinics to deliver ISTp-DP under routine conditions was poor in comparison to IPTp-SP. Several challenges to integration of ISTp with ANC were identified that may need to be considered by countries that have introduced screening at first ANC visit and, potentially, for future adoption of ISTp with more sensitive RDTs. Understanding the effectiveness of ISTp-DP will require additional research on pregnant women’s adherence to ACT.

## Background

Malaria remains a major cause of preventable adverse maternal health and birth outcomes [1, 2]. Each year an estimated > 30 million pregnancies in sub-Saharan Africa are exposed to malaria [3]. Prevention of malaria among pregnant women in this region relies on intermittent preventive treatment with sulfadoxine-pyrimethamine (IPTp-SP) and use of long-lasting insecticide-treated nets [4], however the effectiveness of these strategies is threatened by the development of SP resistance in parasites [5, 6] and insecticide resistance in the vectors [7].

Research efforts to find alternative chemoprevention strategies for the Africa region have intensified over recent years, with mixed results [8]. Six trials to evaluate alternative drugs to replace SP for IPTp showed that amodiaquine [9] and mefloquine [10, 11], were not suitable due to poor tolerability and chloroquine-azithromycin was less efficacious than SP [12]. Four trials explored an alternative prevention strategy of intermittent screening and treatment (ISTp) at every scheduled antenatal care (ANC) visit with malaria diagnosis using a rapid diagnostic test (RDT) and treatment of positive cases with an artemisinin-based combination therapy (ACT) [13–16]. The trials concluded that ISTp with the current generation of malaria RDTs was not a suitable alternative strategy to IPTp-SP, and several trials with new more sensitive RDTs are currently ongoing (ter Kuile, pers. commun.). In addition, four new trials to assess the efficacy, safety and tolerability of dihydroartemsinin-piperaquine (DP) for IPTp are either ongoing (IMPROVE NCT03208179, IMPROVE-2 NCT04158713, NCT02793622) or have been published [17] following promising results from two earlier exploratory trials of IPTp with DP in Kenya and Uganda [15, 18].

The acceptability of both ISTp-DP and IPTp-DP has been assessed in the context of some of these clinical trials [19–23], however the systems effectiveness and uptake of potential alternatives can only authentically be assessed under real life conditions. Replacement of a single dose of IPTp-SP at each ANC visit with more complex and expensive strategies involving multi-day ACT regimens, such as ISTp-DP or IPTp-DP, will be a major determinant of uptake of these interventions once embedded in a national malaria control programme and ultimately their public health impact. However, little is known about their implementation feasibility in this setting.

The study aimed to assess the implementation feasibility of ISTp-DP compared to IPTp-SP in a routine health system setting in western Kenya, conducted in a subcounty adjacent to the site of an open-label three-arm randomized controlled superiority trial comparing IPTp or ISTp with DP to IPTp-SP [15]. This was a mixed methods study and the results of the quantitative component are reported here; the qualitative results are published in a companion paper (Hoyt, pers. commun.).

## Methods

### Study sites and study design

The study was conducted in 12 health facilities in four administrative units (subcounties) in Kisumu County in western Kenya: Muhoroni, Nyakach, Kisumu West and Seme. The study had two phases, an implementation phase which ran for ten months between September 2014 and June 2015, and an evaluation initiated mid-way from February to June 2015. The overall aims of this mixed methods study were to assess implementation feasibility using the framework constructs of Bowen et al*.* [24]: (1) acceptability, (2) demand, (3) implementation, (4) practicality, (5) integration, (6) adaptation and (7) expansion. The constructs of acceptability and demand are addressed in the qualitative study (Hoyt, pers. commun.), while implementation, practicality and integration are addressed directly by both quantitative and qualitative studies (Additional file 1: Table S1). The quantitative study addresses implementation through systems effectiveness analyses including both the overall implementation of each of the interventions, and each component of the interventions. Integration is addressed through exploration of the provision of RDT testing and provision of DP (to RDT test positive cases) by nurses in ANC, whereas in routine practice RDTs are performed by laboratory staff in laboratories, and artemisinin-based combination therapy for treatment of malaria given by clinical doctors. Other constructs are indirectly addressed in the quantitative study including practicality and adaptation and further explored in the qualitative study.

Methods used in the quantitative evaluation reported here comprised health facility audits, observations in ANC clinics, and exit interviews with pregnant women leaving ANC clinics. Health facilities were purposefully selected based on the feasibility of achieving the required sample size within a 3-month period using available ANC records. Three county or subcounty hospitals (level 4 facilities) and three health centres (level 3 facilities) were included in each study arm. The health facilities were first split into two similar groups using health management information systems (HMIS) data on factors likely to have an effect on study outcomes: (1) ratio of ANC registrants per professional staff in ANC, and (2) coverage of one and two doses of IPTp-SP in the previous 12 months. Six matched health facilities were then assigned to one of two arms, ISTp-DP (i.e. the intervention arm) or the current IPTp-SP policy (i.e. the control arm).

The implementation phase began with sensitization and training of Ministry of Health (MOH) staff in the study sites. Members of the County Health Management Team were first sensitized and provided with an overview of the study design. Health facility staff involved in delivering ANC services, including nurses, clinical officers, laboratory staff, data clerks, and pharmacists, were then trained at a central location in each subcounty between August and September 2014. Health staff received a refresher course on Kenyan malaria in pregnancy and related policies, including IPTp-SP, and staff in the ISTp-DP arm received additional training on the use of RDTs, quality control of drugs and RDTs, stock control and storage of RDTs and administration of DP. On completion of training, health staff in the ISTp-DP facilities were provided with standard operating procedures (SOPs) on managing malaria cases, performing RDTs, and administration of DP. DP was supplied to the facilities in the ISTp arm for the duration of the study, and buffer supplies of SP and RDTs of the same brands used by the MOH [namely histidine–rich protein-2 (HRP-2)/Plasmodium lactate dehydrogenase (pLDH) combination RDTs (First Response® Malaria pLDH/HRP2 Combo Test, Premier Medical Corporation Ltd, India)] provided in the event of stockouts in routine MOH supplies. Health facility stocks of anti-malarial drugs (DP and SP) and RDT kits were monitored and replenished when needed to avoid stock-outs, however gloves were not provided. The supply chain, therefore, does not reflect operational realities however other external interference with the study health facilities by study staff such as additional guidance or supervision was kept to a minimum. Respective County and subcounty Health Management Teams were encouraged to continue routine supervision of health facilities and ANC services throughout the study.

Pregnant women attending study health facilities for scheduled focused ANC visits during their second and third trimester received the standard ANC package of care, and either IPTp-SP or ISTp-DP depending on study arm. Pregnant women attending ANC in the IPTp-SP arm received three tablets of quality-assured SP (supplied by Durbin PLC, Middlesex, UK, 500 mg sulfadoxine/25 mg pyrimethamine /tablet) and those in the IPTp-DP arm received a standard 3-day course with DP (Eurartesim®, Sigma Tau, Pomezia, Italy, 40 mg dihydroartemisinin /320 mg piperaquine /tablet). According to national policy [25], women receiving IPTp-SP receive a single dose of three tablets of SP under directly observed therapy (DOT). Women infected with HIV on daily cotrimoxazole are not given IPTp-SP. Since 2010, all health facilities switched to low dose folic acid, with the recommendation for any high dose folic acid to be taken 14 days after a dose of SP [MOH, unpublished]. Women in the ISTp-DP arm who tested RDT positive were prescribed DP based on body weight (24 to 35.9 kg—2 tablets; 36 to 74.9 kg—3 tablets; > 75 kg—4 tablets). Health staff were trained to administer the first dose of DP under DOT and provide the remaining two doses and instructions for how to take them at home. Women with a known HIV positive status were not tested with an RDT unless they were symptomatic for malaria, and if test-positive they were treated following national guidelines for malaria treatment during pregnancy.

### Evaluation procedures

#### Primary endpoints and sample size

The primary endpoint was the “the proportion of eligible women receiving a correctly administered dose of SP, with the 1st dose given by DOT” in the IPTp-SP arm, and the “proportion of eligible pregnant women screened with an RDT and treated according to guidelines” in the ISTp-DP arm. A total of 776 ANC attendees making any visit in second and third trimesters were enrolled for the structured observations and exit interviews, (388 for each intervention). This was a conservative sample size that allowed 10% precision in the outcome estimate with a frequency of 50%, with 95% confidence and a design effect of 2.

Prior to conducting observations and exit interviews at a health facility information about available resources (staffing, services, equipment and stock) and their staff (post, cadre, qualifications, training, access to guidelines) was collected, and other factors which may be associated with uptake of the interventions by pregnant women assessed.

#### Data collection

Prior to the data collection, informed consent was sought from the in-charge of each health facility and key staff working in ANC. During health worker enrollment, information was obtained on professional background, years in post, any recent training and access to malaria guidelines. Pregnant women attending routine ANC were consented once they completed ANC registration and enrolled women were followed throughout their ANC visit by a field worker, from registration until exit from the health facility. Field workers used a structured checklist to assess health provider adherence to study SOPs for prescribing and dispensing practices (SP, DP), proficiency in performing RDTs (ISTp), and adherence to national policy for other routine ANC services. Completion of the checklist was accompanied by collection of unstructured comments on the process to provide context to the findings of structured observations. Exit interviews were then conducted with each consenting woman on completion of the ANC visit. Pregnant women were approached to participate and consented at ANC registration. The selection of pregnant women was based on the availability of field workers, that is, non-probability sampling. On completion of an exit interview, the field worker returned to the registration desk and repeated the process with the next pregnant woman in the queue. Exit interviews included questions on the women’s visit (symptoms or reason for visit, pregnancy history, examinations, tests, drugs given and understanding of how to take them), costs of services received at ANC and any previous hospitalization, and household assets.

### Data analysis

Data were double entered using Epi info. All analyses were adjusted for survey design and clustering of health facilities using STATA 12.1 (StataCorp). Two systems effectiveness analyses were performed for each study intervention and for RDT testing proficiency: (1) cumulative effectiveness and (2) intermediate effectiveness of each process in the delivery chain. For IPTp, intermediate processes assessed comprised: (1) 2nd or 3rd trimester visit; (2) given any SP; (3) given 3 tablets of SP; (4) given SP by DOT; or where was not given IPTp-SP by DOT (5) left the facility with 3 tablets of SP and (6) knows how they would be taken; and (7) a composite endpoint combining 4) and 6). IST intermediate processes were: (1) 2nd or 3rd trimester visit; (2) given an RDT test; (3) RDT negative and not given an anti-malarial; (4) RDT positive and given any DP; (5) given correct dose of DP; (6) given first dose of DP by DOT; (7) Correct number of tablets given; (8) Health worker describes how to take remaining doses; (9) Woman able to repeat instructions; and (10) a composite endpoint combining (3) and (9) [26]. Analyses were restricted to women attending ANC in the second and third trimester, and eligible for either intervention. Analysis was limited to asymptomatic HIV-uninfected participants determined as those who did not receive any antiretroviral during that visit, participants who did not receive any anti-malarials for treatment and for whom information on drugs prescribed during the visit was available on exit. RDT testing proficiency analysis was assessed for 16 steps laid out in the study SOP (listed in Table 6). Univariate analyses were conducted of potential predictors of receipt of: (i) IPTp-SP by DOT vs no DOT; and (ii) ISTp-DP full *vs* incomplete regimen. Logistic regression models adjusted for the survey design were used, and adjusted Wald tests were applied to test for associations between predictors and outcomes. Potential predictors significant at the 10% level (p ≤ 0.1) in the univariate analyses were included in multivariate logistic models to determine which factors were associated with the outcomes after accounting for other potential predictors, and predictors were considered significant at < 0.05 in the final model [26]. A list of potential predictors and the corresponding data source are summarized in Table S2. Principle components analysis (PCA) was used to construct a wealth index in order to assess the effect of socio-economic status (SES) [27].

### Ethics statement

The study was approved by the ethics committees of the Kenya Medical Research Institute’s (KEMRI) Scientific and Ethics Review Unit, Kenya; the Liverpool School of Tropical Medicine, UK; and the London School of Hygiene and Tropical Medicine, UK; the US Centers for Disease Control and Prevention, Atlanta, Georgia, USA relied on KEMRI. Health workers gave signed informed consent for structured questionnaires and for observations. Pregnant women gave signed informed consent for observations and exit interviews at ANC registration. In Kenya, pregnant women aged 15–17 years are considered emancipated minors and were consented directly. Permission from the County Health Management Teams and health facility in-charges was sought prior to initiating the study.

## Results

### Health facility characteristics

Using health management information systems (HMIS) data from 2014, health facilities in each study arm were reasonably well matched with respect to: total catchment population (94,112 and 88,679) and number of ANC visits (2938 and 2454) in the IPTp and ISTp arms, respectively. Health facilities in the ISTp arm had higher numbers of ANC staff (42 versus 21 staff); no data were reported for facility #3 (Table 1). The catchment population for county hospitals ranged from approximately 21,000–23,000, for subcounty hospitals from approximately 10,000–18,000, and health centres from approximately 9000–21,000. Client to staff ratios ranged from 46 to 543, with both extremes found at health centre level. All health facilities had functioning microscopes and all but one health centre had a resident microscopist. All health facilities in the ISTp arm reported using RDTs for malaria diagnosis in the 6 months before the survey with one reporting routine facility stockout of RDTs on the day of the facility audit which was replenished by the study team. One health facility in the ISTp arm reported a stockout of study DP in the 6 months before the survey.

**Table 1:** Characteristics of health facilities in each study arm

Facility type	IPTp-SP arm						ISTp-DP arm					
	1	2	3	4	5	6	7	8	9	10	11	12
	District hospital	District hospital	Sub-district hospital	Health Centre	Health Centre	Health Centre	Sub-district hospital	Sub-district hospital	Sub-district hospital	Health Centre	Health Centre	Health Centre
Level	4	4	4	3	3	3	4	4	4	3	3	3
Catchment population 2014	21,792	22,967	16,622	11,999	9153	11,579	10,480	10,008	18,797	14,311	21,535	13,548
Number of ANC visits 2014	631	679	530	543	230	325	601	280	614	556	NA	403
Number 1st + 4th visits 2014	1010	1107	751	816	320	499	1104	423	1081	818	NA	604
Health staff working in ANC												
Clinical Officer	0	NR	NR	0	1	2	3	2	0	0	0	2
Registered nurse	7	2	NR	1	4	4	4	4	5	3	9	5
Midwife	0	NR	NR	0	0	0	NR	0	5	0	0	0
Total ANC staff reported	7	2	0	1	5	6	7	6	10	3	9	7
ANC clients to staff ratio	90.1	339.5	NA	543	46	54.2	85.9	46.7	61.4	185.3	NA	57.6
ANC tests performed												
Malaria RDT	N	Y	Y	N	N	N	Y	Y	Y	Y	Y	Y
HIV	Y	Y	Y	Y	Y	Y	Y	N	Y	Y	Y	Y
Syphilis	N	Y	Y	N	N	Y	N	N	N	Y	N	N
Hb	N	Y	Y	N	N	Y	N	N	N	Y	N	N
Laboratory												
# functioning microscopes	3	1	2	1	1	1	1	1	1	1	1	3
Resident microscopist	Y	Y	Y	Y	Y	Y	Y	N	Y	Y	Y	Y
RDTs used in last 6 months	N	N	Y	Y	Y	Y	Y	Y	Y	Y	Y	Y
RDTs in stock	NR	NR	Y	N	Y	Y	Y	Y	Y	Y	N	Y
Malaria test conducted in facility	Y	Y	Y	Y	Y	Y	Y	Y	Y	Y	Y	Y
Drug availability												
DP stockout in last 6 months	NR	N	N	N	N	N	N	N	N	N	N	Y
AL stockout in last 6 months	N	Y	N	N	Y	N	Y	N	Y	Y	N	Y
Quinine stockout in last 6 months	N	Y	N	N	Y	N	N	N	NR	Y	N	Y
ITN in stock today	Y	Y	Y	Y	Y	Y	Y	Y	Y	Y	Y	Y
Number of ANC observations + exit interviews	65	65	65	65	65	64	67	64	65	64	63	65

*NA* not available, *NR* not reported

### Health provider characteristics

A total of 112 health providers were interviewed; the mean age was 37 years and 68% were female (Table 2). Approximately one fifth lived in the local catchment area of the health facility where they worked (19%) and approximately one quarter were from the same subcounty as the health facility (28%). Approximately one third of health providers interviewed were registered nurses (35%), 13% were laboratory technicians, 10% were trained HIV counsellors and 8% were clinical officers. Years worked in the health facility ranged from 1 to 5 years (mean 3 years). Only some staff had received training relating to malaria in pregnancy either at a workshop or from a colleague within the last five years, ranging from 20–100% for malaria in pregnancy, 30–94% for case management, 27–50% for RDTs (94% of laboratory staff and 27% of nurses) and 18–78% for ISTp. In general, nurses had greater access to job aids and guidelines than laboratory staff.

**Table 2:** Characteristics of health providers interviewed

Health facility	1	2	3	4	5	6	7	8	9	10	11	12	Overall
Number of staff interviewed	7	11	18	6	4	9	8	11	10	9	11	8	112
Number female	7 (100)	3 (27.3)	12 (66.7)	4 (66.7)	3 (75.0)	7 (77.8)	4 (50.0)	5 (45.5)	9 (90.0)	6 (66.7)	9 (81.8)	7 (87.5)	76 (67.9)
Mean age in years (SD)	33 (7)	35 (10)	39 (8)	37 (11)	46 (10)	31 (4)	34 (5)	37 (9)	36 (7)	42 (9)	36 (9)	41 (12)	37 (9)
Number from village	1 (14.3)	1 (9.1)	5 (27.8)	3 (33.3)	0	4 (44.4)	0	2 (18.2)	1 (10.0)	3 (33.3)	0	2 (25.0)	21 (18.8)
Number from district	1 (14.3)	1 (9.1)	6 (33.3)	1 (16.7)	1 (25.0)	5 (55.6)	1 (12.5)	3 (27.3)	1 (10.0)	4 (44.4)	5 (45.5)	2 (25.0)	31 (27.7)
Ethnicity													
Luo	5 (71.4)	10 (90.9)	13 (72.2)	3 (50.0)	2 (50.0)	9 (100.0)	4 (50.0)	9 (81.8)	5 (50.0)	7 (77.8)	10 (90.9)	6 (75.0)	83 (74.1)
Luyha	1 (14.3)	1 (9.1)	2 (11.1)	0	1 (25.0)	0	2 (25.0)	0	3 (30.0)	0	1 (9.1)	2 (25.0)	13 (11.6)
Kisi	0	0	1 (5.6)	3 (50.0)	1 (25.0)	0	2 (25.0)	2 (18.2)	2 (20.0)	1 (11.1)	0	0	12 (10.7)
Kalenjin	0	0	2 (11.1)	0	0	0	0	0	0	0	0	0	2 (1.8)
Other	1 (14.3)	0	0	0	0	0	0	0	0	1 (11.1)	0	0	2 (1.8)
Mean years in health facility (SD)	5 (5)	3 (3)	4 (3)	5 (4)	2 (1)	4 (4)	3 (2)	1 (1)	3 (2)	5 (3)	4 (3)	3 (3)	3 (3)
Highest qualifications (health facility)													
Medical	0	0	0	0	0	0	1	0	0	0	0	0	1
Clinical Officers	0	3	3	0	0	0	1	1	0	0	1	0	9
Pharmacist	1	1	1	0	0	1	0	0	1	0	0	0	5
Registered Nurse	3	2	3	3	1	2	3	5	4	4	6	3	39
Enrolled Nurse	0	0	1	0	1	0	0	0	1	0	1	2	6
Dispensing technician	0	0	0	0	0	0	0	0	0	0	0	1	1
Lab technician	0	0	0	0	0	1	0	0	0	0	1	0	2
Lab technologist	1	2	1	1	1	1	1	1	2	1	1	1	14
Other technical/pharmaceutical	0	0	0	0	0	0	1	0	0	0	0	0	1
Trained counsellor	1	2	2	0	1	0	0	1	0	2	1	1	11
Other	1	1	7	2	0	4	1	3	2	2	0	0	23
Total staff	7	11	18	6	4	9	8	11	10	9	11	8	112
Number with RDT training last 5 years	3 (42.9)	5 (45.5)	9 (50.0)	2 (33.3)	2 (50.0)	4 (44.4)	3 (37.5)	3 (27.3)	4 (40.0)	3 (33.3)	6 (54.6)	3 (37.5)	42
Number with case management training in last 5 years	6 (85.7)	7 (63.6)	17 (94.4)	5 (83.3)	2 (50.0)	6 (66.7)	6 (75.0)	5 (45.5)	3 (30.0)	3 (33.3)	7 (63.6)	7 (87.5)	66
Number with MiP in training last × years	5 (71.4)	7 (63.6)	17 (94.4)	5 (83.3)	4 (100.0)	8 (88.9)	8 (100.0)	9 (81.8)	2 (20.0)	5 (55.6)	7 (63.6)	6 (75.0)	74
Number with IST training last × years	2 (28.6)	2 (18.2)	5 (27.8)	4 (66.7)	1 (25.0)	3 (33.3)	4 (50.0)	5 (45.5)	2 (20.0)	7 (77.8)	7 (63.6)	5 (62.5)	42
Daily access to guidelines (MiP, diagnosis, RDTs, case management)	4 (57.1)	6 (54.6)	14 (77.8)	5 (83.3)	3 (75.0)	8 (88.9)	7 (87.5)	8 (72.7)	5 (50.0)	8 (88.9)	9 (81.8)	5 (62.5)	73
Daily access to job aides (MiP, diagnosis, RDTs, case management)	5 (71.4)	5 (45.5)	13 (72.2)	5 (83.3)	3 (75.0)	7 (77.8)	6 (75.0)	7 (63.6)	7 (70.0)	9 (100.0)	8 (72.7)	6 (75.0)	72

### Pregnant women characteristics

A total of 777 pregnant women were observed during an ANC visit on the day of the survey and interviewed as they exited the health facility, 389 in the IPTp arm and 388 in the ISTp arm (Table 3). There were approximately equal numbers of women in the second and third trimester in both arms: 51% and 49%, respectively, in the IPTp arm (mean gestation 6.4 months), and 49% and 51%, respectively, in the ISTp arm (mean gestation 6.5 months). Similarly, there was an approximately equal distribution of women attending for their first, second, third, fourth or more (4+) ANC visit in both arms, with slightly more women attending for their first visit in the IPTp arm (29%) and for their second visit in the ISTp arm (27%) compared to the other visits (not statistically significant). Over 40% of women in each arm were multigravidae aged between 20 to 24 years. Most women were married, 78% and 82% in the IPTp and ISTp arms respectively, and of Luo ethnicity, 88% and 89%, respectively. Most women had primary level education (55% and 53%, respectively), and approximately one fifth had no education (30% and 24%, respectively). Over 70% of women had at least one child aged under 5 years, and 11–12% had had a child who died.

**Table 3:** Characteristics of pregnant women in ANC observations and exit interviews

Socio-demographic data	IPTp-SP (N = 389)		ISTp-DP (N = 388)	
	n	% (95% CI)	n	%(95% CI)
Trimester^a^				
2nd trimester	195	50.9 (40.6, 61.2)	188	49.2 (45.1, 53.3)
3rd trimester	188	49.1 (38.8, 59.4)	194	50.8 (46.7, 54.9)
Mean months gestation		6.4 (IQR 5–8)		6.5 (IQR 5–8)
ANC visit no				
1	113	29.1 (22.2, 37.0)	97	25.0 (19.3, 31.8)
2	92	23.7 (18.4, 29.9)	104	26.8 (22.1, 32.1)
3	97	24.9 (21.1, 29.2)	90	23.2 (16.7, 31.3)
4+	87	22.4 (18.4, 26.9)	97	25.0 (17.6, 34.2)
Malaria symptoms/fever				
Yes	6	6.6 (4.03, 10.61)	5	7.0 (1.90, 22.85)
No	85	93.4 (89.39, 95.97)	66	93.0 (77.15, 98.10)
Gravidity				
Primigravidae	98	25.2 (20.36,30.73)	112	28.9 (23.95, 34.33)
Secundigravidae	110	28.3 (25.93,30.75)	102	26.3 (20.59, 32.92)
Multigravidae	181	46.5 (41.58,51.55)	174	44.9 (42.02, 47.71)
Median number of births		1.62 (1.57,1.68)		1.6 (1.57, 1.69)
Age group (15–49)				
15 to 19 years	70	18.0 (14.55, 22.05)	71	18.3 (13.79, 23.87)
20 to 24 years	163	41.9 (38.36, 45.53)	163	42.0 (36.32, 47.92)
25 to 29 years	92	23.7 (19.18, 28.79)	92	23.7 (20.35, 27.44)
30+ years	64	16.5 (13.33, 20.14)	62	16.0 (11.44, 21.88)
Median age	23	IQR 20–28	23	IQR 20–27
Marital status				
Married	310	79.7 (74.59, 83.99)	319	82.2 (77.07, 86.41)
Divorced/widowed	61	15.7 (10.92, 22.00)	65	16.8 (12.69, 21.79)
Single	18	4.6 (2.76, 7.67)	4	1.0 (0.36, 2.94)
Education level ±				
None/nursery	115	29.8 (22.50, 38.28)	91	23.58 (19.83, 27.78)
Primary	214	55.44 (51.80, 59.03)	205	53.11 (47.77, 58.37)
Secondary	36	9.33 (6.57, 13.09)	63	16.32 (10.93, 23.67)
College/university	21	5.44 (2.39, 11.91)	27	7.00 (4.30, 11.17)
Ethnic group				
Luo	344	88.43 (79.41, 93.81)	346	89.2 (80.69, 94.20)
Luyha	28	7.2 (3.14, 15.64)	28	7.2 (3.11, 15.87)
Kalenjin	7	1.8 (0.30, 9.93)	6	1.6 (0.37, 6.22)
Kisii	6	1.5 (0.27, 8.19)	3	0.8 (0.31, 1.94)
Other	4	1.0 (0.36, 2.91)	5	1.3 (0.46, 3.53)
Has 1+ child aged < 5 years	206	70.8 (61.84, 78.37)	201	72.8 (66.3, 78.50)
Has lost a child/child died	36	12.4 (10.70, 14.27)	29	10.5 (7.01, 15.45)

^a^11 missing; ± 5 missing

### Systems effectiveness of ANC to deliver IPTp-SP

Of 336 women included in the analysis, 75% (239/319) received a dose of SP and all received the correct number of tablets, i.e. 3 tablets, however, only 76% (179/237) of these received the dose by DOT (Table 4). This translates into a cumulative effectiveness for receiving IPTp-SP by DOT of 57%. The cumulative effectiveness for the composite endpoint (doses given with or without DOT) was 53.6%.

**Table 4:** Intermediate and cumulative systems effectiveness of delivery of IPTp-SP (any dose)

IPTp-SP arm (N = 342)^a^	N	n	Intermediate process effectiveness, % (95% CI)	Cumulative delivery effectiveness %
DOT				
1. 2nd or 3rd trimester^b^	342	336	100	
2. Given any SP^c^	319	239	74.9 (54.8,88.0)	74.9
3. Given dose of 3 tablets	239	237	100	74.9
4. Took dose by DOT	237	179	75.5 (31.7,95.4)	56.5
With or without DOT				
1. Given any SP	319	239	74.9 (54.8,88.0)	74.9
2. Given dose of 3 tablets^d^	239	237	100	74.9
3a. Took dose by DOT	237	179	75.5 (31.7,95.4)	56.5
3b. SP not given by DOT	237	58	24.5 (4.6,68.3)	24.5
4. Has SP on exit	58	2	3.4 (0.2,38.0)	3.4
5. Knows how to take 3 tablets SP^e^	2	1	100.0 (0,100)	3.4
6. Composite endpoint	336	180		53.6

^a^Analysis limited to participants who were not given any antimalarial for treatment and/or antiretroviral and for whom information on drugs received was available at exit

^b^6 missing

^c^17 missing

^d^2 missing

^e^1 missing

### Systems effectiveness of ANC to deliver ISTp-DP

Of 347 women included in the analysis, 90% (289/320) were tested with a malaria RDT of whom 11% (32/289) tested positive (Table 5). Of women who tested positive, 91% (29/32) were given DP of whom 86% (25/29) were given the correct dose based on the woman’s weight, with a cumulative effectiveness of 71%. Of the women receiving the correct dose, only 44% (11/25) received the initial dose of DP by DOT and only 18% (2/11) of these received instructions from the health provider on how to take the remaining doses. Neither of these women were able to repeat these dosing instructions on exit resulting in 0% cumulative effectiveness for women who tested positive. The overall cumulative effectiveness for the composite endpoint was 60.8%. The proportion of women screened with an RDT was relatively consistent across visit number (1–4+ visits), with slightly fewer women screened with an RDT at a first visit as these women have blood slides taken as part of ANC profile testing (20% [11.4, 33.9]) compared to 4+ visits (27% [17.7, 38.1]). More women attending their first ANC visit tested positive for malaria (4% [1.2, 9.6]) than women attending for 4+ visits (2% [0.5, 5.9]) (not significant).

**Table 5:** Intermediate and cumulative systems effectiveness of delivery of ISTp-DP

IST arm (N = 360)^a^	N	n	Intermediate process effectiveness, % (95% CI)	Cumulative delivery effectiveness, %
1. 2nd or 3rd trimester women	347	347	100	100
2. Given an RDT test^b^	320	289	90.3 (70.7, 97.3)	90.3
3a. RDT negative	289	257	88.9 (84.3, 92.3)	90.3
3b. RDT −ve not given any drug	211	211	100	90.3 (3b = tested − ve)
4a. RDT +ve	289	32	11.1 (7.7, 15.7)	90.3
4b. RDT+ ve given any DP	32	29	90.6 (59.8, 98.4)	81.8
5. DP given at correct dose^c^	29	25	86.2 (56.5, 96.8)	70.5
6. Given stat dose by DOT	25	11	44.0 (15.0, 77.7)	31.0
7. Correct no. tablets taken	11	11	100	31.0
8. Health worker describe to woman how to take remaining doses	11	2	0	5.6
9. Woman able to repeat instructions	2	0		0 (9 = tested+ ve)
10. Composite endpoint	347	211		60.8

^a^Analysis limited to participants who were not given any antiretroviral and who did not report having a fever

^b^27 missing

^c^DP dose based on weight, stat dose given by DOT and remaining two doses given to take home—24 to 35.9 kg, 2 tablets; 36 to 74.9 kg, 3 tablets; > 75 kg, 4 tablets

### RDT proficiency among ANC and laboratory staff

Of the 289 women screened with an RDT in the ISTp arm, a higher proportion of tests were performed in ANC (95%, 274/289) than in the lab (Table 6). Cumulative effectiveness for adherence to each RDT proficiency step in ANC was 12% (35/289) if step #5, selecting the fourth finger to prick for blood (65%, 82/126), and step #7, labelling the RDT with the patient’s name (only 22%, 26/117 were labelled), are omitted. The omission of these two steps is based on the assumptions that: i) the finger used to collect blood does not affect the test result; ii) in this setting the woman is given the result during the consultation by the nurse during her ANC visit thereby removing the need to label the RDT. Two further steps with low adherence to the SOP in ANC were the wearing of gloves (47%, 129/274), which is important for health worker safety, and waiting time for the test result (30%, 35/117), which may affect accuracy of test results. Cumulative effectiveness for steps 1–15 of tests performed in the lab was higher than in ANC (36% vs 12% without steps 5 and 7, respectively), the steps with lowest adherence being wearing gloves (73%, 11/15), selecting the fourth finger (36%, 4/11), and waiting time for the test result (55%, 6/11). Steps where ANC nurses performed better than lab staff were selecting the fourth finger (percentage score difference of+ 29) and using guidelines (+ 11).

**Table 6:** RDT proficiency among laboratory and ANC staff in health facilities in the ISTp-DP arm

Intermediate process	Lab			ANC			
	n	Intermediate process effectiveness	Cumulative delivery effective-ness	n	Intermediate process effective-ness ±	Cumulative delivery effective-ness ≠	Difference (Intermed-iate processes), %
		% (95% CI)	%		% (95% CI)	%	
RDT performed							
1. 2nd and 3rd trimester^a^	289	90.3 (70.7, 97.3)	90.3	289	90.3 (70.7, 97.3)	90.3	0
2. RDT performed ANC or lab	15	5.2 (0.6, 34.7)	90.3	274	94.8 (65.3, 99.4)	90.3	0
3. Gloves worn	11	73.3 (34.9, 93.4)	66.2	129	47.1 (13.8, 83.2)	42.5	-26.2
4. RDT opened in front woman	11	100	66.2	126	97.7 (78.1, 99.8)	41.5	-2.3
5. Fourth finger selected	4	36.4 (1.5, 95.6)	24.1	82	65.1 (51.1, 76.9)	27	+ 28.7
6a. Finger cleaned with alcohol	4	100	24.1	79	96.3 (76.4, 99.5)	26	-3.7
6b. Without step 5	11	100	66.2	117	92.9 (76.9, 98.1)	40	-7.1
7. RDT labelled patient’s name	10	90.9 (0.0, 100.0)	60.2	26	22.2 (10.5, 41.1)	8.9	-68.7
8a. Correct volume/ml blood added	10	100	60.2	26	100	8.9	0
8b. Without step 7	11	100	66.2	117	100	40	0
9. Blood added to correct place	11	100	66.2	117	100	40	0
10. Buffer added after blood	11	100	66.2	117	100	40	0
11. Buffer added correct place	11	100	66.2	117	100	40	0
12. > 20 < 35 min waiting time	6	54.5 (0.7, 99.5)	36.1	35	29.9 (13.7, 53.4)	12	− 24.6
13. Control line visible	6	100	36.1	35	100	12	0
14. RDT result read accurately	6	100	36.1	35	100	12	0
15. Result given to women	6	100	36.1	35	100	12	0
16. Guidelines used	0	0	0	4	11.4 (0.3, 83.4)	1.4	+ 11.4

^a^Includes 6 missing trimester

### Predictors of women receiving IPTp-SP by DOT

Predictors of women receiving SP by DOT in the univariate analysis were marital status, sick visit (as reported by or agreed with the woman), paid for a lab test, and being given drugs at the pharmacy (Table 7). In the final multivariate model, only payment for a lab test was independently associated with receipt of a dose of SP by DOT (AOR6.43, 95% CI 2.07,19.98).

**Table 7:** Predictors of systems effectiveness—IPTp-SP by DOT

Determinant	n	%	OR	95% CI	P value	AOR	95% CI	P value
Marital status					0.07			0.07
Single	36	63.2	1			1		
Married	139	51.3	0.61	0.24, 1.54		0.28	0.10, 0.81	
Divorced/widowed	4	28.6	0.23	0.08, 0.65		0.55	0.01, 42.23	
Sick visit					0.103			
No	143	54.6	1					
Yes	36	45.0	1.47	0.89, 2.41				
Payment for lab test					0.05			0.01
No	23	76.7	1			1		
Yes	5	38.5	5.26	1.02, 27.13		6.43	2.07, 19.98	
Drugs given in pharmacy					0.01			
No	176	58.09	1					
Yes	3	18.75	6.0	1.87, 19.29				

### Predictors of women receiving ISTp-DP

Predictors of women being screened with an RDT in the univariate analysis were: marital status, gravidity, education, SES, urban or rural location, consultation time, consultation duration, sick visit (reported by the woman), the presence of a resident microscopist at the facility, and delivery of prevention-of-mother-to-child-(HIV) transmission (PMTCT) services (Table 8). In the final multivariate model, married women (AOR 0.77, 95% CI0.47,1.26), and women living in rural areas (AOR 0.78, 95% CI 0.02,0.27) were less likely to receive ISTp-DP while women making a sick visit (AOR 1.78, 95% CI 1.31,2.41) and being tested in a health facility with a resident microscopist (AOR 3.75, 95% CI 1.31,2.40) were more likely to receive ISTp-DP.

**Table 8:** Predictors of systems effectiveness—ISTp-DP

Determinant	n	%	OR	95% CI	P value	AOR	95% CI	P value
Gravidity					0.06			
Primigravidae	89	42.4	1					
Secundigravidae	80	37.7	0.82	0.50, 1.35				
Multigravidae	120	33.8	0.69	0.50, 0.97				
Marital status					0.01			0.009
Single	53	42.1	1			1		
Married	234	37.2	0.82	0.49, 1.36		0.77	0.47, 1.26	
Divorced/widowed	2	9.1	0.14	0.04, 0.46		0.13	0.04, 0.43	
Education level^a^					0.1			
Primary	155	37.0	1					
Secondary	49	49.5	1.67	1.06, 2.62				
College/university	19	39.6	1.11	0.52, 2.38				
None/nursery	65	31.6	0.78	0.48, 1.28				
SES					0.1			
1 most poor	43	26.5	1					
2	56	37.1	1.63	0.82, 3.23				
3	53	34.2	1.44	0.83, 2.49				
4	72	46.8	2.43	1.25, 4.72				
5 least poor	65	41.9	2.0	0.62, 6.42				
Urban/rural					< 0.00019			0.0009
Urban	56	83.6	1			1		
Rural	233	32.8	0.096	0.03, 0.30		0.78	0.02, 0.27	
Consultation time					0.08			
a.m	76		1					
p.m	100		1.70	0.93, 3.12				
Consultation duration					0.026			
< 1.5 h	173		1					
> 1.5 h	6		0.23	0.11, 0.84				
Sick visit					0.0007			0.0015
Yes	47	29.0	1			1		
No	242	39.4	1.59	1.28, 1.97		1.78	1.31, 2.41	
Resident microscopist					0.07			0.05
Yes	250	35.1	1			1		
No	39	60.9	2.90	0.89, 9.43		3.75	1.31, 2.40	
PMTCT services given					0.13			
Yes	1	4.2	1					
No	242	36.67	13.31	1.21, 146.20				

## Discussion

This is the first implementation feasibility study of a ‘screen and treat’ strategy undertaken in sub-Saharan Africa despite several clinical trials to assess its potential as an alternative preventive strategy to IPTp-SP [13–16]. The study found that *implementation* as assessed through the systems effectiveness of ANC clinics to deliver ISTp-DP according to guidelines in a routine setting was poor and highlights several challenges for *integration* in ANC. The study provided useful insights for countries in sub-Saharan Africa that have introduced an *adaptation* of ISTp which uses screening at first ANC visit alongside IPTp as a ‘hybrid strategy’ [28] and for potential adoption of ISTp with more sensitive RDTs. The delivery of IPTp-SP, the current policy in Kenya since 1999 [29], performed better but was nevertheless imperfect. These results for IPTp-SP are similar to previous observational studies measuring the systems effectiveness of ANC clinics to deliver IPTp-SP through routine health systems in Kenya [30], with adherence to DOT in both the Kenya studies exceeding that observed in Mali [26]. Healthcare provider performance may be improved through targeted interventions combining training, supervision, and group problem solving [31], and would be most effective if done simultaneously with the introduction of any new intervention to avoid the persistent challenges and pitfalls experienced with IPTp-SP.

The ISTp-DP strategy not only requires the delivery of more components than IPTp-SP, making it more complex and time consuming to deliver, it includes the administration of ACT with a 3-day regimen that has so far only been recommended and used for treatment of clinical cases [32]. The use of ACT for the prevention of malaria in pregnancy is, therefore, a new drug indication, and this study was the first time that health providers will have used DP for IPTp under routine conditions and that ANC nurses had administered an artemisinin-based combination within ANC clinics. The complexity and novelty of the ISTp-DP intervention was reflected in the lower cumulative effectiveness of women who tested positive compared to IPTp-SP and highlighted several *practical issues* that would need to be addressed if delivery of ISTp were to be integrated in ANC.

*Integration* of ISTp in ANC will require a substantial role change for ANC staff involving malaria testing, drug administration and counselling. The practice of nurses performing malaria RDTs within ANC clinics was a new practice, with pregnant women routinely referred to laboratories to have malaria diagnosis performed by laboratory technicians. Nurses do, however, routinely perform HIV testing and counselling. While there were some obvious differences in the RDT proficiency between staff from ANC and the laboratory, this analysis revealed that ANC staff are capable of conducting RDTs comparatively well and consistently across ANC visits 1–4 + . An earlier study in this region of Kenya [33] using microscopy showed a prevalence of 18% in pregnant women attending ANC in the second and third trimesters. RDTs are known to have lower sensitivity than microscopy in pregnant women which may explain the lower prevalence (11%) found in this study. Alternative possible explanations are reducing trends in malaria transmission or provider proficiency with RDTs.

Key departures from the study SOP among ANC staff, such as not wearing gloves, labelling the test with the client’s name, or waiting for the full test time before reading the result, may be an indication of time constraints within ANC. This would be consistent with the qualitative findings where ANC nurses reported that delivering ISTp-DP increased their workload (Hoyt, pers. commun.), however there was no association between receipt of ISTp-DP and client-to-staff ratio in the logistic regression analysis. The study did not provide gloves, and it is possible that gloves were not routinely provided to nurses working in ANC. Regarding labelling of RDT tests, the test result and drug (if test-positive) were given at the point of care such that nurses may not have perceived the need to label tests, in contrast to tests done on symptomatic patients in the laboratory where treatment is prescribed by clinical staff located elsewhere in an outpatient department. Indeed, the systems effectiveness analysis showed that a high proportion of women who tested positive were given DP during the ANC consultation. Regarding the test wait time, while the test result can appear earlier than the recommended wait time, training on use of RDTs should emphasise that adhering to wait times is critical to ensure accuracy. *Integration* of RDT tests in ANC may, therefore, be possible, however staff proficiency in using RDTs would need to be periodically reassessed to ensure quality as part of routine supervision. The views and perceptions of laboratory, pharmacy and nursing staff on these role changes are explored in-depth in the qualitative study which showed that nurses were generally enthusiastic about taking on these roles, whereas laboratory and pharmacy staff voiced concerns over nurses performing testing and drug administration, respectively.

With regards to *integration* of the administration of DP, even though over two thirds of women were given the correct dose according to weight by nursing staff, less than half these women were given the first dose by DOT of whom less than a fifth comprehended the instructions on how to take the remaining doses at home. The analysis shows that the requirement to give IPTp and the first dose of ISTp by DOT has a high impact on systems effectiveness. Data on adherence to SP given to pregnant women to take at home is sparse placing even greater emphasis on the importance of assuring health provider adherence to DOT for IPTp-SP. Findings from the paired qualitative study suggest that women often refuse to take SP by DOT on an empty stomach for fear of side effects such as nausea (Hoyt, pers. commun.) and other studies have shown that health providers may potentiate this reaction by first asking women whether they have eaten before giving IPTp-SP by DOT [34, 35].

Adherence to DP when used for prevention in pregnancy was key concern voiced by health providers in the acceptability study, highlighting that information about the timing of doses must be easy for women to comprehend, with emphasis on the importance of completing the full course [19]. There is a weak evidence base on adherence to multi-day ACT regimens for treatment of malaria in other populations [36], and apparently no evidence on adherence in pregnant women.

The qualitative component of the feasibility study and also the acceptability study conducted in the trial setting found that the delivery of ISTp-DP by ANC nurses was viewed positively by most health providers as it reduces wait times for women and improves continuity of care [19]. The qualitative study found the main implementation challenges for ISTp-DP reported by laboratory staff was the perceived lack of RDT sensitivity in comparison to microscopy, which they thought may leave women unprotected and, therefore, vulnerable to malaria, a concern not shared by ANC nurses [19]. Indeed, the clinical trial comparing ISTp-DP and IPTp-DP versus IPTp-SP showed ISTp-DP to be inferior to IPTp-SP in this area of high SP resistance and high malaria transmission [15]. The authors of this and the other ISTp trials concluded that at the current levels of RDT sensitivity [13, 14, 16], ISTp with RDTs for testing is not a suitable alternative to IPTp-SP in areas of high SP resistance.

In an attempt to bolster malaria in pregnancy prevention strategies some countries in sub-Saharan Africa with high levels of SP resistance have already adopted an *adaptation* of ISTp by combining single test and treatment at first ANC visit as a ‘hybrid strategy’ with IPTp-SP, using RDTs in ANC in Tanzania [37] and microscopy or RDT in Kenya (Kariuki, pers. commun.). Since adopting this hybrid strategy, the uptake of routine testing as an ANC-based intervention in Tanzania has increased substantially from 36.7% in 2014 to 88.8% in 2017 [37]. Furthermore, recent modelling studies using trial data from Kenya and five other countries (Burkina Faso, The Gambia, Ghana, Mali and Malawi) have shown that a hybrid strategy may offer additional benefit especially in areas with high-grade SP resistance [38]. The authors of the modelling paper suggest that screening and treatment in the first trimester in areas where IPTp-SP is contraindicated, for example in areas of very high SP resistance, could also substantially improve pregnancy outcomes, particularly if using highly sensitive RDTs.

This study highlights several *practical* issues that will need to be addressed if ISTp were to be integrated in ANC. These include, for example, formal role changes to enable nurses to take on test and treat strategies, changes to supply and management procedures for drugs and RDTs, training on RDT testing and drug administration and counselling, and expansion of supervision checks on nurses. Screening for malaria as part of integrated point-of-care testing for malaria at ANC with other standard tests routinely provided to pregnant women such as HIV, syphilis, and malaria, has shown promise. A study in western Kenya by Young et al*.* [39] showed that integrated testing led to a substantial increase in testing rates for syphilis, malaria, and anaemia, providing an opportunity for improved management of all four conditions in the context of a well-established HIV rapid testing programme in pregnancy. The study found that high staff turnover was a predictor of pregnant women not receiving a full antenatal screening profile at first visit, it did not however assess health provider proficiency of the four RDT tests. In a related qualitative study, high client to healthcare worker volume ratio, stock-outs and poor working conditions challenged the delivery of adequate counselling and management of the four conditions [40].

## Limitations

The study had several limitations. While ANC client to staff ratio was a criterion for matching health facilities however the number of staff and ANC attendance rates changed between designing the study and undertaking the evaluation (18 months later). However, the logistic regression analysis for predictors did not find an association between receipt of IPTp or ISTp and either consultation duration or ANC staff to client ratio, though there was an association between consultation duration and ISTp receipt in the univariate analysis. Efforts were made not to interfere with routine MOH health system practices, but some level of study oversight was required to ensure a constant supply of RDTs and study drugs. The unstructured observations used for identifying systems effectiveness may have been influenced by the Hawthorne effect, where participants who are being observed may change their usual behaviour or practices [41, 42]. In this case, it was assumed that any change in health provider behaviour was towards ‘best behaviour’ but the sample size/study duration was considered sufficient for any behaviour change to revert back to normal during the course of the study [40]. Also, interpretation of the findings suggest that the Hawthorne effect was an unlikely factor given low systems effectiveness, particularly of ISTp-DP [43]. The process for enrolling pregnant women was based on feasibility and was not technically random, such that there may be a bias towards selection of women attending at certain times of day. Data on the timing of doses of IPTp-SP was obtained from ANC cards and the IPTp-SP analysis restricted to doses taken at least one month apart. Finally, stock availability on the day of the survey is assumed on the basis on the monitoring reports from the study coordinator, and no stockouts were reported during the survey days.

## Conclusion

The systems effectiveness of ANC clinics to deliver the ISTp-DP under routine conditions was poor as was the delivery of IPTp-SP, a far simpler strategy to deliver yet still presenting implementation challenges more than two decades after its introduction. The study highlights several practical issues that would be needed for integration of ISTp-DP and provides useful insights for countries in sub-Saharan Africa that have introduced the ‘hybrid strategy’ and for any future adoption of ISTp with more sensitive RDTs. RDT testing by ANC staff is feasible and simple proficiency testing may be used to identify areas that require strengthening. The administration of DP by ANC nurses however appears to present a challenge, particularly provision of the first dose by DOT and the quality of patient counselling. Evidence for pregnant women’s adherence to multi-day ACT regimens for either prevention or treatment will be needed to assess overall effectiveness of the ISTp strategy.

## Supplementary information

**Additional file 1: Table S1.** Thematic framework for feasibility analysis and corresponding sources of data. **Table S2.** Potential predictors of effectiveness of health service delivery of IPTp-SP or ISTp-SP.

## Data Availability

The datasets during and/or analysed during the current study available from the corresponding author on reasonable request.

## Abbreviations

ANC: Antenatal clinic
DOT: Directly observed therapy
DP: Dihydroartemisinin-piperaquine
IPTp: Intermittent preventive treatment in pregnancy
SP: Sulfadoxine-pyrimethamine
RDT: Rapid diagnostic test
