# Supplementary material for: Intermittent screening and treatment with dihydroartemisinin-piperaquine for the prevention of malaria in pregnancy: implementation feasibility in a routine healthcare system setting in western Kenya
Source: Malar J. 2020 Nov 25;19:433. doi: 10.1186/s12936-020-03505-0 (PMC7690090; doi:10.1186/s12936-020-03505-0)
Supplement: Supplementary file 1 — Additional file 1: Table S1. Thematic framework for feasibility analysis and corresponding sources of data. Table S2. Potential predictors of effectiveness of health service delivery of IPTp-SP or ISTp-SP. [file 12936_2020_3505_MOESM1_ESM.docx]

**Additional file**

**Table S1. Thematic framework for feasibility analysis and corresponding sources of data***

| **Theme** | **Description** | **Study method** |
| --- | --- | --- |
| **Acceptability** | To what extent is a new idea, programme, process or measure judged as suitable, satisfying, or attractive to programme deliverers? And perceived as such to users by deliverers? | IDIs |
| **Demand** | To what extent is a new idea, programme process, or measure likely to be used (how much demand is likely to exist)? | FGDs |
| **Implementation** | To what extent can a new idea, programme, process, or measure be successfully delivered to intended participants in some defined, but not fully controlled context? | OBS and ExI  IDIs |
| **Practicality** | To what extent can a new idea, programme, process, or measure be carried out with intended participants using existing means, resources, and circumstances without outside intervention? | OBS and ExI  IDIs |
| **Integration** | To what extent can a new idea, programme, process, or measure be integrated within an existing system? | IDIs; OBS and ExI |
| **Adaptation** | To what extent can a new idea, programme, process, or measure be or be likely to be implemented as designed? | OBS; ExI; IDIs |
| **Expansion (scale-up)** | To what extent can a new idea, programme, process, or measure be expanded to provide a scaled-up programme? | OBS; ExI; IDIs |

IDIs: Indepth interviews with health providers; FGDs: focus group discussions with pregnant women; OBS: observations in ANC; ExI: exit interviews with pregnant women

Adapted from Bowen *et al.* [24]

**Table S2. Potential predictors of ineffective processes of health service delivery of IPTp-SP or ISTp-SP**

|  | **IPTp-SP** | **ISTp-DP** | **Data source*** |
| --- | --- | --- | --- |
| **ANC client factors** |  |  |  |
| Gravidity | Y | Y | 1self-report |
| Trimester | Y | Y | 1self-report, 2derived from LMP |
| Number of children <5 yrs | Y | Y | 1self-report |
| Age | Y | Y | 1self-report |
| Marital status | Y | Y | 1self-report |
| Education | Y | Y | 1self-report |
| Socio-economic status | Y | Y | 1self-report |
| **Visit related factors** |  |  |  |
| Consultation time | Y | Y | 1 recorded |
| Consultation duration | Y | Y | 1 recorded |
| ANC visit no. | Y | Y | 1self-report, 2 |
| Has an illness | Y | Y | 1self-report |
| Symptomatic for malaria | Y | Y | 1self-report |
| Malaria test result | Y | Y | 1recorded, 2 |
| Payment for malaria test |  | Y | 1self-report |
| Other payment | Y | Y | 1self-report |
| Non-malaria illness | Y | Y | 1self-report |
| Side effects to medicines given today | Y | Y | 1 self-report |
| Had previous test for malaria | Y | Y | 2 |
| Confirmed malaria on previous visit | Y | Y | 2 |
| Place where RDT is done |  | Y | 1 recorded |
| Place where drugs are given | Y | Y | 1 recorded |
| PMCTC | Y | Y | 1 recorded |
| HIV test | Y |  | 1 recorded |
| Physical exam | Y | Y | 1 recorded |
| **Health facility factors** |  |  |  |
| Type of health facility | Y | Y | 1, 3 |
| Health facility | Y | Y | 1, 3 |
| Client to staff ratio | Y | Y | 3 |
| Resident microscopist |  | Y | 3 |
| **Health provider factors** |  |  |  |
| Cadre of HW performing RDT |  | Y | 1 recorded, 4 |
| Highest professional qualification |  | Y | 4 |
| Years in post |  | Y | 4 |
| Training on Mip | Y | Y | 4 |
| Training on RDTs |  | Y | 4 |
| Training of IST | Y |  | 4 |
| Daily access to MiP guidelines | Y | Y | 4 |
| Daily access to RDT guidelines |  | Y | 4 |
| Daily access to diag guidelines |  | Y | 4 |
| Daily access to CM guidelines |  | Y | 4 |

*1-Observation checklist and Exit interview (recorded or self-report); 2-ANC card; 3-health facility audit; 4-health worker enrollment form
